# Induction Heating of Laminated Composite Structures with Magnetically Responsive Nanocomposite Interlayers for Debonding-on-Demand Applications

**DOI:** 10.3390/polym16192760

**Published:** 2024-09-30

**Authors:** Eleni Gkartzou, Konstantinos Zafeiris, Christos Tsirogiannis, Alberto Pedreira, Adrián Rodríguez, Pablo Romero-Rodriguez, Giorgos P. Gakis, Tatjana Kosanovic-Milickovic, Apostolos Kyritsis, Costas A. Charitidis

**Affiliations:** 1 Research Lab of Advanced, Composite, Nano-Materials and Nanotechnology (R-NanoLab), School of Chemical Engineering, National Technical University of Athens, 9 Heroon Polytechniou, GR-15780 Zografou, Greece; egartzou@chemeng.ntua.gr (E.G.); kzafeiris@chemeng.ntua.gr (K.Z.); christsiro@chemeng.ntua.gr (C.T.); tkosanovic@chemeng.ntua.gr (T.K.-M.); 2 AIMEN Technology Center, 36418 O Porriño, Spain; 3 Dielectrics Group, Physics Department, School of Applied Mathematical and Physics Science, National Technical University of Athens, 9 Heroon Polytechniou, GR-15780 Zografou, Greece

**Keywords:** induction heating, debonding on demand, magnetic nanoparticles, 3D printing, additive manufacturing, CFRP, PAEK, disassembly, magnetically responsive, nanocomposite

## Abstract

In the present study, the feasibility to achieve localized induction heating and debonding of multi-material composite structures is assessed in testing coupons prepared by Automated Fiber Placement (AFP) and extrusion-based additive manufacturing (AM) technologies. Nano-compounds of Polyether-ketone-ketone (PEKK) with iron oxide nanoparticles acting as electromagnetic susceptors have been processed in a parallel co-rotating twin-screw extruder to produce filament feedstock for extrusion-based AM. The integration of nanocomposite interlayers as discrete debonding zones (DZ) by AFP-AM manufacturing has been investigated for two types of sandwich-structured laminate composites, i.e., laminate-DZ-laminate panels (Type I) and laminate-DZ-AM gyroid structures (Type II). Specimens were exposed to an alternating magnetic field generated by a radio frequency generator and a flat spiral copper induction coil, and induction heating parameters (frequency, power, heating time, sample standoff distance from coil) have been investigated in correlation with real-time thermal imaging to define the debonding process window without compromising laminate quality. For the optimized process parameters, i.e., 2–3 kW generator power and 20–25 mm standoff distance, corresponding to magnetic field intensities in the range of 3–5 kA m^−1^, specimens were effectively heated above PEKK melting temperature, exhibiting high heating rates within the range of 5.3–9.4 °C/s (Type I) and 8.0–17.5 °C/s (Type II). The results demonstrated that localized induction heating successfully facilitated debonding, leading to full unzipping of the debonding zones in both laminate structures. Further insight on PEKK nanocomposites debonding performance was provided by thermal, morphological characterization and non-destructive inspection via X-ray micro-computed tomography at different processing stages. The developed framework aims to contribute to the development of rapid, on-demand joining, repair and disassembly technologies for thermoplastic composites, towards more efficient maintenance, repair and overhaul operations in the aviation sector and beyond.

## 1. Introduction

Thermoplastic (TP) composites are becoming increasingly popular in the aviation sector and constantly expanding their range of use for both primary and secondary structures, exhibiting high damage tolerance, ease of formability, production efficiency, cost-effectiveness, lenient storage conditions, long shelf life and recyclability [1]. Automated composite manufacturing technologies employing high-performance TP polymers, namely the polyaryl ether ketones (PAEK) group of polymers (polyether ether ketone—PEEK, polyether ketone ketone—PEKK, Low Melt-PAEK), PPS (polyphenylene sulfide) and PEI (polyetherimide), enable strong bonding, flexibility in design, homogeneous stress distribution, repair and reprocessing potential, and fatigue resistance [2]. These benefits are crucial in designing high-performance components that are lightweight and meet the stringent safety and sustainability requirements in the aerospace industry.

As more TP composite structures are manufactured, robust joining strategies need to be developed to ensure parts can be assembled efficiently at scale. Polymer welding is characterized by the heating of joining parts, bringing them into intimate contact and promoting the diffusion of polymer chains across the interface [3]. Since polymers are generally poor heat conductors, internal heat generation mechanisms, such as resistance, induction and ultrasonic welding, have been extensively investigated for the joining and repair of TP parts, preventing the formation of large heat-affected zones [4,5]. In this respect, notable examples of new assembly technologies employed by the aerospace industry include the assembly of the leading edges of the wings of the Airbus A340-600 and A380 by resistance welding, and induction welding applied on the empennage of the Gulfstream G650, resulting in significant process advancement and extensive certification programs with full-scale component tests [6]. In parallel, the development of new techniques and processes for the easy recycle and repair of bonded structures is becoming of great interest for the industry, with a growing need to develop adhesives and joining methods that maintain their adhesion strength during service life but can be easily dismantled upon the application of external stimuli for repair, reuse, or recycling [7].

Induction heating (IH) is a non-contact, energy-efficient process to generate localized heat with precise temperature control, speed, reproducibility, and adaptability to complex geometries. IH relies on the heating of electromagnetic and conductive materials (susceptors) placed within an alternating electromagnetic field operating in the kHz to MHz frequency range [8]. A typical IH system consists of a radio frequency (RF) power generator connected to an external circuit for impedance matching and a set of capacitors connected in parallel to an induction coil, allowing to reach the targeted resonance frequency, which produces a time-variable magnetic field in its surroundings. The magnetic field intensity distribution is influenced by power, frequency, coil geometry and coil-to-workpiece electromagnetic coupling (i.e., standoff distance from the workpiece surface), thus the combined effect of these factors should be investigated to define an optimum processing window, i.e., achieve internal heat conduction and temperature uniformity without overheating and material degradation [9,10].

The main electromagnetic dissipation phenomena that can be exploited for IH include Joule heating due to eddy currents, hysteresis heating and relaxation losses [11]. For ferro/ferri-magnetic materials in multi-domain magnetic state, the reversal of magnetization direction takes place via magnetic domain wall displacement, and a small amount of energy is dissipated during each magnetic polarization–depolarization cycle (hysteresis loop) [12]. As magnetic hysteresis is a non-equilibrium process, the theoretical maximum amount of heating power is rarely realized due to the occurrence of relaxation processes [13]. In the case of particulate susceptors below a critical particle size (superparamagnetic limit), the multi-domain state becomes energetically unfavorable, and each particle represents a single magnetic domain with superparamagnetic behavior, exhibiting strong magnetization along the direction of the external magnetic field. However, the reduced particle volume and associated decrease in the energy barrier against magnetization reversal act in favor of relaxation losses [14]. In addition, the degree of agglomeration has also been found to induce weak dipole–dipole interactions, leading to agglomerates with hysteretic heating behavior [15]. In this context, only certain combinations of particle size, size distribution, external field frequency and amplitude may fully exploit IH potential [13].

Due to their unique properties, high-surface-to-volume ratio, tunable reactivity and enhanced functionality, nanoparticles, including magnetic, are increasingly shaping a wide range of industries and becoming an integral part of various everyday products and technologies, offering highly specialized and effective solutions (e.g., drug delivery systems, hyperthermia treatment, advanced imaging techniques to smart coatings, remediation, magnetic data storage, catalysis) to complex challenges in precision engineering [16,17,18]. Polymer systems with magnetic nanofillers relying on hysteresis losses for heat generation are considered promising for attaining uniform and efficient heating at lower concentrations [19]. The amount of heat generated per mass unit of magnetic material and per unit time is frequently reported as the Specific Absorption Rate (SAR) and is affected by several intrinsic and extrinsic factors, including particle shape, size, chemical composition, agglomeration state and external magnetic field intensity and frequency [20,21]. By selecting magnetic particles with a paramagnetic transition above the polymer melting temperature range, a self-regulating heating process can be achieved for controlled heating applications [22,23]. Various types of magnetic particles, including iron and iron oxide-containing ferrites, nickel and cobalt–nickel alloys have been investigated as powder additives to provide inductive heating functionality, with recent studies further contributing to the fundamental understanding of their unique micro-/nanoscale magnetic properties [24,25,26]. To improve the inductive heating characteristics of magnetic nanostructures, many approaches have been taken to investigate the SAR correlation with particle size, composition, shape, inter-particle interaction and inter-phase exchange coupling, while synthetic strategies have been proposed to obtain engineered nanoparticles with precise control over their magnetic properties [24].

In polymer systems, several studies have presented new susceptor configurations with embedded magnetic micro-/nanoparticles, with a special focus on the development of film susceptors for structural adhesive joints. The induction heating behavior of thermoplastic polyurethane (TPU) adhesive film with embedded iron oxide (Fe_3_O_4_) nano-/microparticles (0.27, 2, and 9 μm average size) was examined by Bae et al., considering the effect of particle size, concentration and susceptor film thickness, with heating rates up to 3.1 °C/s and maximum temperature up to 324.5 °C (t = 500 s) [27]. The amount of heat generation was found to be proportional to the content of Fe_3_O_4_ particles, film thickness and input power, while an inverse effect demonstrated with the increase of Fe_3_O_4_ particle size. In a similar study, TPU susceptors with iron particles (8, 43 and 74 μm average size) were assessed, with composites consisting of larger iron particles demonstrating a higher heating rate (up to 2.7 °C/s for 20 wt% Fe) in all compositions tested [28]. On the contrary, in a study conducted by Baek et al., the maximum temperature reached (200 °C or higher within t = 10 s) and did not present a significant dependency on the Fe_3_O_4_ weight ratio for film susceptors (450 μm thickness) prepared by mixing polyamide 6 and Fe_3_O_4_ (average size: 200 nm) at different ratios (50, 67, 75 and 80 wt% of Fe_3_O_4_) [29]. In a recent study by Raczka et al., the influence of different Fe_3_O_4_ agglomeration states (dispersed, micrometer-assemblies, hard agglomerated) incorporated in a polydimethylsiloxane (PDMS) matrix was investigated [14]. For a field amplitude above coercivity threshold, a larger hysteresis surface area and higher heating rate were attained for composites with dispersed nanoparticles, while an identical heating performance was recorded at lower field amplitudes. This effect was attributed to the possible contribution of additional force of neighboring nanoparticles in magnetic moment relaxation mechanisms, also indicated by a decrease in coercivity and remanence in agglomerated states. 

Furthermore, particles of iron, nickel and magnetite have been evaluated as susceptor materials in polypropylene (PP) and PEEK matrices by Martin et al., complemented with an analytical model for the prediction of the heating capacity [19]. Susceptor samples were prepared by melt mixing magnetic particles with either PP or PEEK at 5 and 10% vol. compositions. Fe_3_O_4_ was found to be more suitable for PEEK heating, with the 10% vol. PEEK/Fe_3_O_4_ sample exhibiting temperature increase above 400 °C after 45 s, while a moderate heating efficiency was recorded for the 5% vol. PEEK/Fe_3_O_4_ sample, which reached a plateau temperature of 283 °C, in equilibrium with thermal losses in the surrounding media. Cheng et al. investigated the induction heating performance and adhesive strength of poly(ethylene-methacrylic acid) (EMAA) adhesives with different mass loading (5, 20 and 30 wt%) of Fe_3_O_4_ nanoparticles (50–100 nm diameter) [30]. Nanocomposite adhesives with 5 wt% Fe_3_O_4_ were found to be effective in retaining 100% of the original bond strength for up to five cycles, measured using a tensile lap shear test. A limited number of thermoplastic compounds have been commercialized, e.g., proprietary compounds developed by Emabond Solutions, USA, available in several formats (extruded profiles, injection molded gaskets, AM feedstock) that can generally be applied as additional materials in the weld zone for the formation of the tongue-in-groove type of shear joints or custom joint configurations [22].

Material extrusion AM is particularly versatile regarding material compatibility and multi-material integration, spanning different polymer types and functionalities. Additive manufacturing enables local control over material properties, and this, if coupled with multi-material integration, can be used to create joints with tailored physical and mechanical adhesive properties, opening the possibility to explore new joint design strategies. Tailoring AM adherends and adhesives, following Design for AM (DfAM) principles has shown great promise, with multiple investigations in the field of adhesives applied to AM components [31]. Recently, extrusion-based AM has been employed to embed circuits and heating elements for joining by the application of a controlled electric current [32]. Strategies for AM joining include functionally graded structures that modify the adherend geometry and compositional variations to change material properties for a tunable adhesive stiffness [33]. In parallel, AM offers several advantages to apply Design for Disassembly (DfD) principles. Using a multi-material AM approach in combination with thermally expandable microspheres, easily separable compounds have been achieved using heat as the external trigger [34]. Magnetically active composites as raw materials for extrusion-based AM have also been assessed, demonstrating that the coupling of filler loading with applied magnetic field frequency and intensity can be employed for the proper control of heating capacity [35]. Yet additional research is required to enhance manufacturing reliability and repeatability and further expand to new material types and applications [31].

Considering the above, the aim of this study is to assess the feasibility of the integration of discrete debonding zones in multi-material composite structures to achieve targeted thermal activation and the separation of CFRP laminates for debonding-on-demand applications. To this end, a previously investigated nano-compound of PEKK with Fe_3_O_4_ nanoparticles has been processed to produce filament feedstock for extrusion-based AM [36]. An integrated manufacturing process sequence of AFP/AM was employed for the fabrication of sandwich-structured composite laminates with magnetically responsive interlayers, which were subsequently exposed to an alternating magnetic field to assess their induction heating performance and define the debonding process window without compromising laminate quality. Further insight on PEKK nanocomposites debonding performance was provided by thermal, morphological characterization and non-destructive inspection via X-ray micro-computed tomography at different processing stages. The developed framework is part of an innovative data-driven methodology to design, manufacture and maintain multi-functional and intelligent airframe parts through a cost-effective, flexible and multi-stage manufacturing system based on the combination of robotized AFP and Fused Filament Fabrication (FFF) technologies, developed in the framework of H2020 DOMMINIO project (G.A. No. 101007022) [37].

## 2. Materials and Methods

### 2.1. FFF Nanocomposite Feedstock Preparation

The production of the nanocomposite filament was performed via compounding and filament extrusion process by employing a co-rotating parallel twin-screw extruder (Thermo Fisher Scientific Process 11, Karlsruhe, Germany) equipped with a gravimetric feeding system to introduce nanoparticles in powder form and a melt pump coupled with the extruder setup to stabilize extrudate diameter for monofilament production, according to the methodology described in previous study [36]. The take-up setup consisted of an air-cooling system (conveyor belt-based), a triaxial laser system (ODAC 13TRIO, Zumbach Electronic AG, Orpund, Switzerland) for real-time inspection of filament diameter and ovality, and a winding system operating with a synchronized spooling rate. The PEKK material employed in this study was a medium flow grade PEKK copolymer with 60/40 ratio of terephthaloyl to isophthaloyl moieties and low crystallization rate (KEPSTAN^®^ 6002, Arkema, Dove, France, Europe). Nanoparticles of Fe_3_O_4_ with average diameter of 20 nm with Polyvinylpyrrolidone (PVP) coating (1% content) and 99.5% purity were supplied by GetNanoMaterials, France. Before processing, feedstock materials were dried at 120 °C for 6 h. The nanocomposite composition used in this study (7.5 wt% content of Fe_3_O_4_ nanoparticles) was selected based on previous research, where a comparative assessment of different nanoparticle types and concentrations in PEKK matrix was conducted to assess induction heating efficiency in specimens produced by injection molding [36]. Extrusion conditions for nanocomposite filament production are presented in Table 1. Nanocomposite filament with 7.5 wt% content of Fe_3_O_4_ nanoparticles was produced with average diameter of 1.79 ± 0.11 mm.

### 2.2. Additive Manufacturing of Sandwich Composite Specimens 

Robot-assisted AFP and FFF processes were employed for composite specimen manufacturing through an integrated, multi-stage manufacturing workflow (Table 2). Two types of sandwich-structured composite laminate panels were prepared, i.e., laminate-debonding zone-laminate panels (Type I Specimens), consisting of two monolithic composite laminates manufactured by AFP and an FFF interlayer of nanocomposite filament with magnetic susceptors in the middle, and laminate-debonding zone-FFF gyroid (Type II Specimens), with an FFF gyroid structure (pure PEKK) 3D printed on top of the debonding zone. 

Continuous fiber, monolithic composite laminates were manufactured by AFP employing high-performance LM-PAEK unidirectional (UD) prepreg tapes with 66% carbon fiber mass fraction (Cetex TC 1225, Toray Advanced Composites). The in situ consolidated laminates were composed of unidirectional layers, following a stacking sequence of [45/0/135/0/0/135/90/45/0]s and [−45/45/0/0/90/90/45/−45]s for Type I and Type II specimens, respectively. Optimum process parameters were defined to maximize interlayer adhesion and minimize the void content, namely a 6 kW diode laser was used to reach a nip point temperature of 400 °C while applying 500 N compaction force, 250 mm/s layup speed and 220 °C mold temperature. After AFP manufacturing, the top surface of the composite laminate was heated by the laser source above LM-PAEK melting temperature to promote adhesion during FFF deposition of the nanocomposite filament with magnetic susceptors. The FFF process focused on obtaining the optimum quality parameters during deposition, using a 0.8 mm nozzle with a nominal layer height of 0.3 mm, extrusion width of 0.8 mm, 10 mm/s printing speed and nozzle temperature of 370 °C. In Type I specimens, a top composite laminate with the same stacking sequence as described above was manufactured by AFP over the nanocomposite layer. In Type II specimens, a gyroid lattice structure with unit cell size of 15 mm and a top solid layer was 3D printed via FFF using commercial PEKK filament (ThermaX™ PEKKKA, 3DXTECH, Grand Rapids, MI, USA). Composite panels of 250 × 250 mm were subsequently cut with a water jet cutter to obtain testing coupons with equivalent active surface areas (2000 mm^2^) for the assessment of induction heating efficiency. 

### 2.3. Characterization Methods

#### 2.3.1. Thermal Analysis

Differential Scanning Calorimetry (DSC) was employed to study the specimens’ nanocomposite layer (PEKK and 7.5% Fe_3_O_4_ NPs) in three different stages of thermal processing, namely pellet form (Stage 1—S1), FFF nanocomposite layer extracted from Type I specimens (Stage 2—S2) and remelted nanocomposite after being subjected to induction heating and extracted from the debonded specimens (Stage 3—S3). The calorimetric measurements were performed employing a TA Q200 DSC apparatus (TA Instruments, New Castle, DE, USA), calibrated with sapphire for heat capacity and indium for temperature and enthalpy on samples of ~6–9 mg in mass closed in TA standard aluminum pans. The specimens were initially heated at a rate of 10 °C/min from room temperature to 360 °C for 5 min. This temperature value was selected since it is above the PEKK equilibrium melting temperatures to erase the thermal history. Afterwards, specimens were cooled at a rate of 40 °C/min to room temperature, followed by a second heat scan at a rate of 10 °C/min up until 400 °C. The second heat scan enabled the measurement of the glass transition and melting temperatures, and the melting enthalpy for specimens obtained from the three processing stages (S1–S3). Thermogravimetric analysis was carried out with a NETZSCH/STA 449 F5 Jupiter thermal analysis system under a synthetic air atmosphere from 25 °C to 900 °C and a heating rate of 10 °C/min to investigate the thermal stability and the effect of thermal processing stages (S1–S3) at the onset of thermal degradation. To this end, the onset decomposition temperatures corresponding to 5% weight loss of the initial mass were calculated from each TGA thermogram, denoting the temperature at which thermal decomposition begins.

#### 2.3.2. Scanning Electron Microscopy (SEM)

Surface analysis of debonded laminate specimens was performed using SEM coupled with Electron Diffraction Spectroscopy (EDS). SEM characterization was conducted in a Hitachi TM3030Plus SEM to study the morphology of the nanocomposite layer (PEKK and 7.5% Fe_3_O_4_ NPs) and perform elemental analysis, following the debonding of the tested specimens. All specimens were sputter-coated with gold to effectively observe the morphological details of the debonded areas.

#### 2.3.3. Micro-Computed Tomography (mCT)

Segments derived from nanocomposite FFF filament, Type I and II specimens were analyzed with micro-computed X-ray tomography with SkyScan 1272 High Resolution Micro-CT (Bruker microCT, Kontich, Belgium) for the assessment of nanoparticle degree of agglomeration and non-destructive inspection of Type I and II specimens. The obtained shadow angular projections were used for the reconstruction of the virtual slices through the sample. Raw data cross sections were generated using NRecon reconstruction software (v1.7.0.4 by Bruker microCT) by implementing the Feldkamp algorithm. The original grayscale slices were processed in CT-Analyser (v1.18.4 by Bruker microCT) to improve detail resolution (contrast enhancement) and proceed with particle isolation and segmentation. Reconstructed results were visualized as a set of orthogonal slices crossed at selected points of the reconstructed volume in DataViewer software (v1.2.5.7 by Bruker microCT). Morphological analysis in filament samples was conducted by sampling sub-volumes of interest of 5 mm^3^ to reduce computation time.

### 2.4. Induction Heating and Thermal Imaging

#### 2.4.1. Induction Heating Setup

Induction heating of Type I and II specimens was performed using a TruHeat HF 5010 unit (Trumpf Hüttinger, Freiburg, Germany) with max. 10 kW power supply, generator current up to 35 A and frequency range of 50 kHz to 1000 kHz. The RF generator is connected to the external circuit, comprising a 16:1 transformer (560 A current transformer output) and 4 × 0.33 μF capacitors, forming a series circuit together a flat spiral inductor made of two concentric ellipsoid turns, connected to the output. The application of an alternating voltage induces a periodic oscillation of current and voltage in the series circuit, where the frequency is automatically calculated by the generator to reach the LC resonance frequency of the induction coil. To investigate the induction heating capacity of the embedded debonding zones in Type I and Type II specimens, RF power supply values within the range of 2–3 kW were tested in conjunction with different coupling distances (standoff distance) between the induction coil and the workpiece in the range of 20–45 mm. Induction heating process parameters are summarized in Table 3.

**Figure 1:**
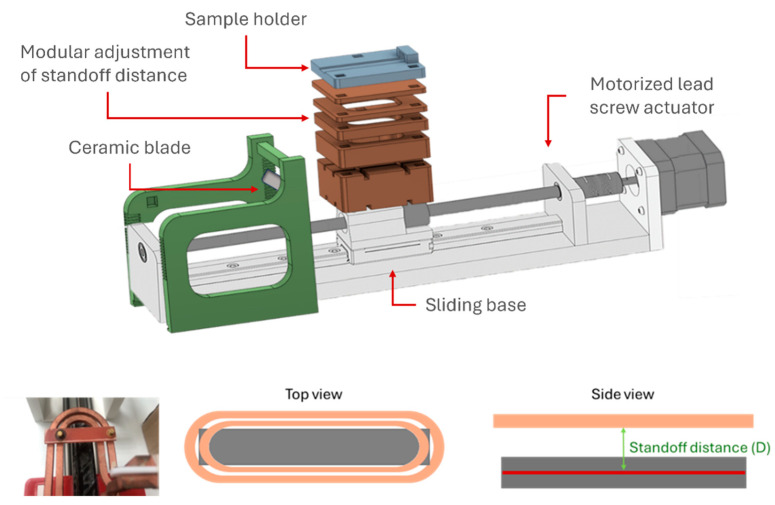
Schematic of the mounting setup for specimen static heating and debonding trials.

**Figure 2:**
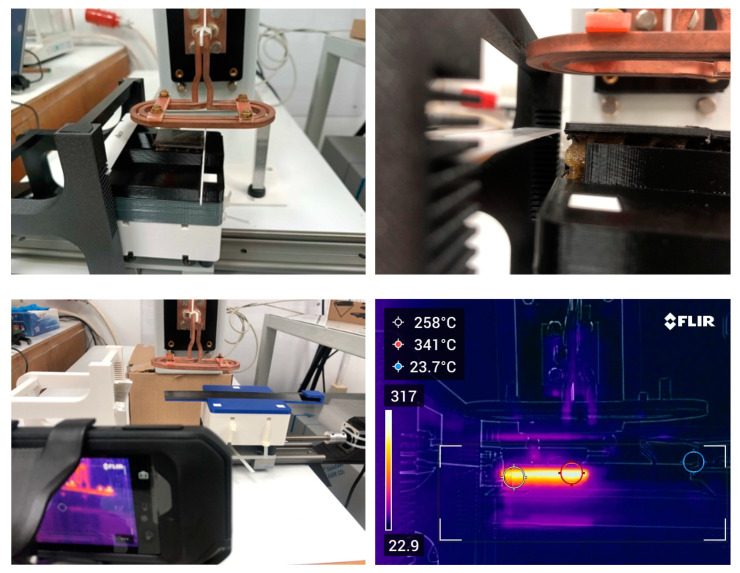
Induction heating testing setup.

For specimen static heating and debonding trials, a bespoke mounting setup was designed and 3D printed (Figure 1 and Figure 2). The mounting setup consisted of a linear axis with a motorized lead screw actuator for specimen movement below the coil, a sliding base with modular subcomponents secured in place with 3 alignment pins to adjust the standoff distance and a ceramic blade mounting base consisting of two mirrored columns with sequential 1 mm slots for adjustment of blade height. Specimen temperature was recorded throughout the entire experiment with the use of a thermal imaging camera (FLIR C5, Teledyne FLIR LLC., Wilsonville, OR, USA).

#### 2.4.2. Induction Heating Simulation

**Figure 3:**
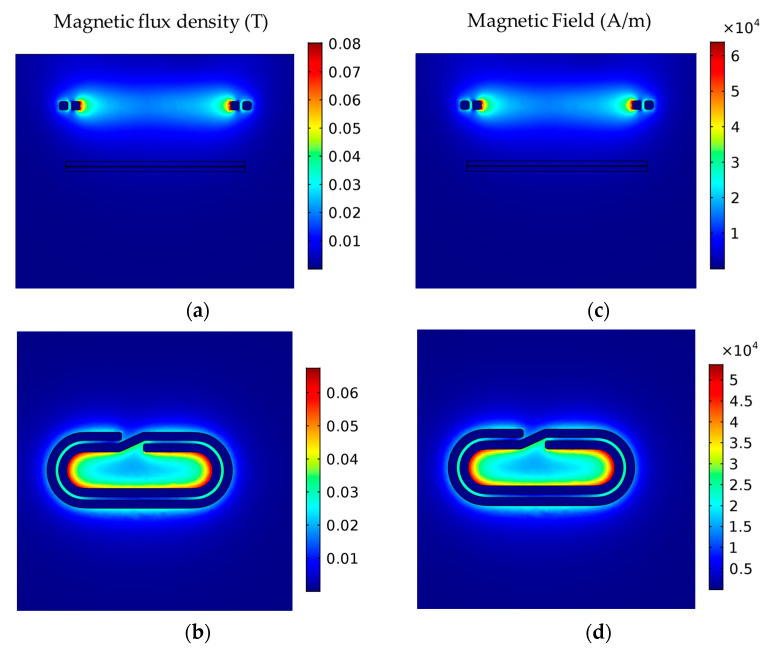
Simulation results for magnetic flux density norm (**a**,**b**) and magnetic field intensity (**c**,**d**) for 30 mm standoff distance (top/side coil view).

**Figure A1:**
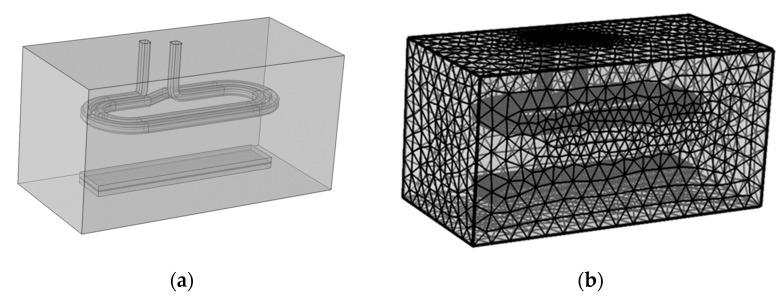
(**a**) Simulated geometry for the induction heating model; (**b**) computational mesh used to discretize the computational domain.

A computational model was developed to simulate the induction magnetic field generated in the surroundings of the coil during the induction heating process. A computational domain was designed to simulate the induction heating setup (Figure A1), including the geometrical characteristics of the flat spiral/pancake coil and the dimensions of the workpieces. The pancake-ellipsoid coil was simulated by imposing the respective experimental conditions of Table 3. A computational mesh of 197,247 elements was used to discretize the computational domain, and a quadratic basis function was employed for the dependent variables. The developed model is based on previous works, and the set of equations (as described in Appendix A) was solved using COMSOL Multiphysics software v5.5 [38,39,40,41]. As in previous works, a bounding box around the experimental setup was selected to model the surrounding air, and a magnetic insulation boundary condition was imposed on the bounding box boundaries. The size of the bounding box size was determined by increasing the box size until the model results were unaffected by further size increase. Indicative simulation results for magnetic flux density norm and magnetic field intensity for 30 mm standoff distance are presented in Figure 3.

## 3. Results and Discussion

### 3.1. Filler Dispersion Analysis

**Figure 4:**
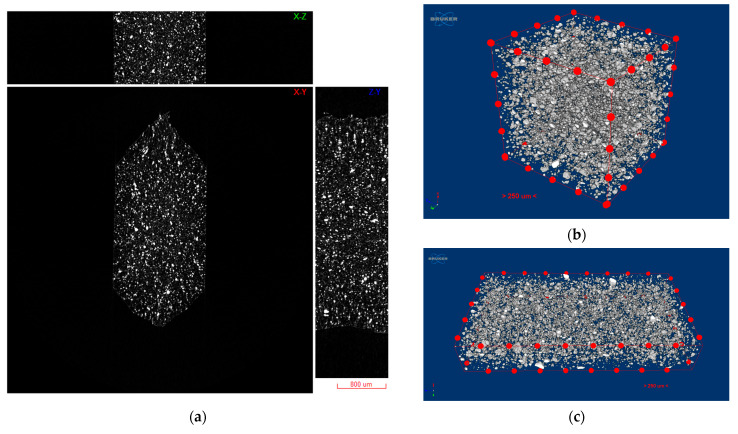
Indicative images of agglomerate distribution for PEKK + Fe_3_O_4_ 7.5 wt% FFF filament sample: (**a**) XZ, XY, ZY cross sections of reconstructed grayscale slices (scale bar: 800 μm); (**b**,**c**) 3D visualization of sample volume (scale bar: 250 μm, 2.5 μm voxel size).

The reconstructed results were initially visualized as a set of three orthogonal slices crossed at selected points of the reconstructed volume in DataViewer software (version 1.2.5.7 by Bruker microCT). The nanoparticle agglomerates in FFF filament are highlighted as high X-ray absorption areas (towards white in grayscale), while the surrounding polymer matrix is depicted in darker gray (Figure 4a). Ambient air surrounding the samples is depicted as black.

**Figure 5:**
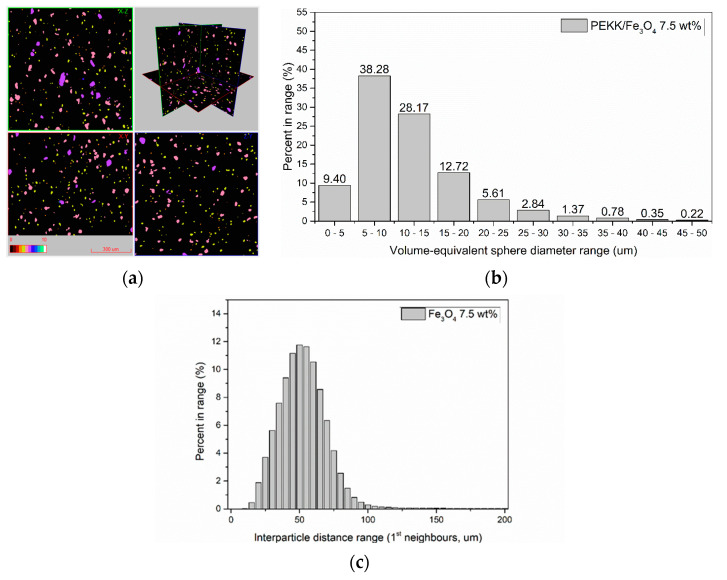
(**a**) Color-coded 2D visualization of agglomerate size (scale = 300 μm); (**b**) agglomerate size classification employing the volume-equivalent sphere diameter model (PEKK + Fe_3_O_4_ 7.5 wt%); (**c**) distribution of agglomerate separation distances for PEKK + Fe_3_O_4_ 7.5 wt%.

Due to the high nanoparticle concentration and the inherent agglomeration tendency of magnetic nanoparticles, the mixing process during compounding was not able to eliminate agglomerated particles, as observed in Figure 4b,c. Nonetheless, an even dispersion of agglomerates was obtained, which was further analyzed to assess the agglomerate size distribution and average distances. To this end, agglomerated particles were labeled by colors corresponding to their size, grouped in size classes of 5 μm range (namely 0–5 | 5–10 | … | >45 μm), and the volume-equivalent sphere diameter model was employed for agglomerate size classification. The accuracy of this classification approach is considered sufficient, given the average sphericity values for all datasets investigated were above 0.84. The mean separation distance between each agglomerate and its nearest neighbor was also assessed by a 3D construction process obtained through a Delaunay triangulation algorithm [42]. For this analysis, a custom python script was developed to post-process the list of centroid coordinates of each agglomerate particle, as extracted from the individual object analysis processed by CT-Analyser (v1.18.4 by Bruker microCT). Centroid coordinates were analyzed to identify agglomerate particles in close proximity (first neighbors) and subsequently build a 3D Delaunay triangulation mesh connecting the respective centroid points. The mesh edge lengths connecting first neighbors were subsequently calculated and plotted to derive the distribution of agglomerate separation distance. As observed in Figure 5b, the majority of agglomerates (>88%) are below 20 μm diameter range, with a mean diameter of 12 ± 7 μm. In addition, an even distribution of agglomerates is confirmed from the calculated interparticle distances, which are centered around 50 μm average distance (Figure 5c). 

### 3.2. Thermal Properties

**Figure 6:**
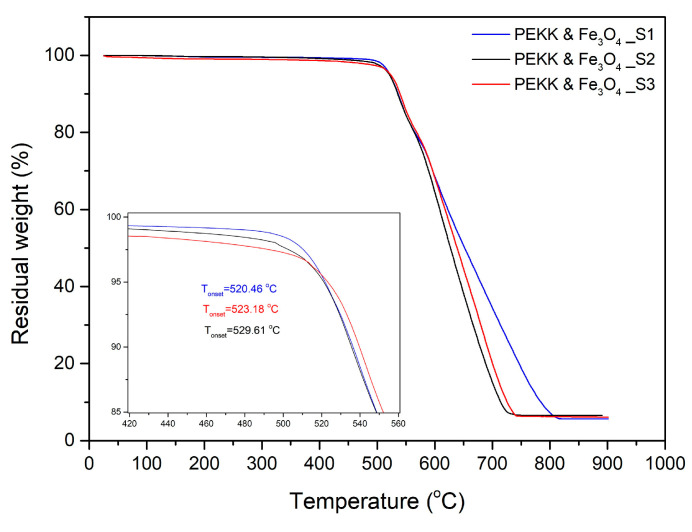
Comparative TGA thermograms for each processing stage, namely pellet form (S1), FFF interlayer (S2), after re-melting with induction heating (S3).

TGA thermograms and calculated values of thermal degradation onset temperature (T_o_) for each stage are presented in Figure 6. The results indicated the materials to be stable up to at least 500 °C without significant degradation. A small increase is observed for the onset of thermal degradation between the nanocomposite material in pellet form (T_o_ = 520.5 °C) and the subsequent processing stages (529.6 °C and 523.2 °C for FFF and re-melted material, respectively).

As reported in the literature, PEKK matrices can evolve due to the chemical transformation of the macromolecular chains, possibly attributed to crosslinking mechanisms initiated by scissions located in the carbonyl and ether bonds, creating radicals that miss hydrogen molecules. Radicals can then rearrange by removing hydrogen molecules from aromatics cycles forming phenyl radicals, which can then rearrange with an adjacent radical to produce crosslinks [43]. Another possibility for phenyl radical is to rearrange by internal combination to produce dibenzofuran or fluorenone derivatives. In this context, the increase in thermal stability observed between S1 and S2 processing stages may be related to crosslinking mechanisms activated during FFF processing.

**Figure 7:**
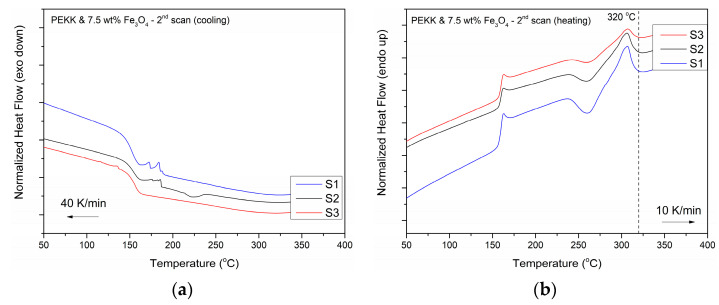
DSC thermograms for each processing stage, namely pellet form (S1), FFF interlayer (S2), after re-melting with induction heating (S3). (**a**) Cooling cycle; (**b**) 2nd scan heating cycles.

Figure 7 represents the DSC thermograms for each processing stage. The plots present the second heating and cooling cycles employed for the calculation of thermodynamic quantities. From this analysis, the nanocomposites have a glass transition temperature (T_g_) of 159 °C, which is the same as the value reported for the pure pseudo-amorphous PEKK matrix [44]. The addition of magnetic nanoparticles has a moderate nucleation effect in the polymer matrix, with cold crystallization peaks appearing at 261–263 °C for all processing stages, followed by melting peaks at 306–307 °C (Table 4). The melting region for all samples tested was found below 320 °C, which was set as the minimum temperature required to achieve debonding during induction heating.

### 3.3. Coupon-Level mCT Inspection

**Figure 8:**
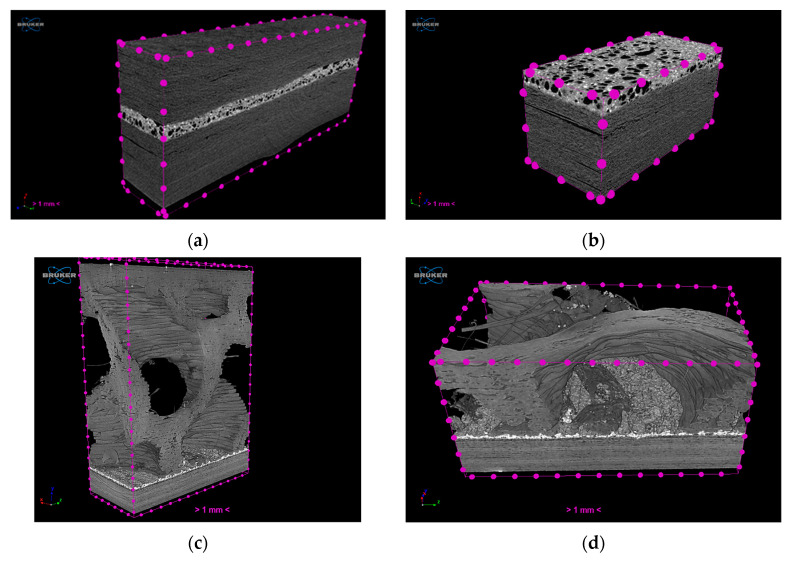
Three-dimensional visualization of Type I (**a**,**b**) and Type II (**c**,**d**) specimens and FFF nanocomposite interlayer before induction heating (white regions: FFF interlayer and NP agglomerates, gray region: CFRP/polymer matrix, black: background/air; scale bar: 1 mm).

The assessment of the internal structure of composite laminate specimens was conducted via mCT scanning. A reference sample was scanned before treatment at a 5.5 μm voxel size. Top/bottom CFRP laminates have a high compaction degree with minimum defects. In the case of the FFF nanocomposite layer, a high degree of porosity was observed for Type I specimens, with variable pore and NP agglomerate sizes (Figure 8a,b). In comparison with mCT analysis conducted in Section 3.1, where no porosity was observed in specimens of the same NP concentration obtained from FFF filament samples (Figure 4), it may be derived that this effect is introduced during the FFF process. Nonetheless, as far as the debonding functionality of the nanocomposite material is concerned, there was no significant variation during induction heating for the parameters tested, thus indicating that the effect of porosity, if any, was the same for all specimens investigated. An improved compaction and uniformity of the FFF nanocomposite layer was obtained for Type II specimens, as it can be observed in Figure 8c,d.

### 3.4. Induction Heating Capacity Assessment

#### 3.4.1. Induction Simulation

**Figure 9:**
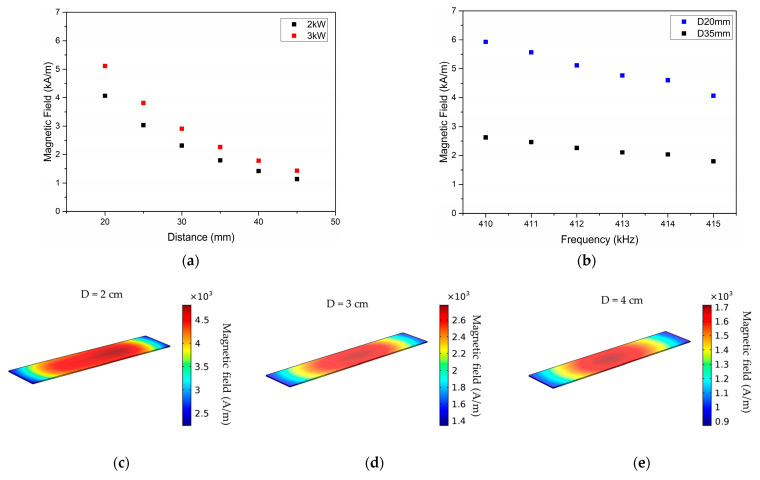
Absolute value of the induced magnetic field intensity (H) simulated for different standoff distances (**a**) and generator power/frequency (**b**) settings; (**c**–**e**) magnetic field intensity simulated for the FFF debonding zone located at different standoff distances from the coil.

The absolute value of the induced magnetic field intensity (H) was simulated for the FFF debonding zone, considering different standoff distances away from the coil and different generator power/frequency settings, as shown in Figure 9a,b. Based on the defined induction process parameters (Table 3) and simulation results, magnetic field intensities in the range of 1–5 kA m^−1^ were experimentally tested. The effect of generator power on magnetic field intensity becomes more pronounced with decreasing standoff distance (Figure 9a), with up to 25% higher intensity simulated for 3 kW power in comparison with 2 kW for a 20 mm standoff distance. When comparing the high/low levels of standoff distance for different frequencies (calculated automatically by the RF generator by the defined power setpoints, Table 3), it is confirmed that the workpiece to coil distance has a prominent contribution to the magnetic field intensity (Figure 9b). With the increasing coil distance from the FFF debonding zone, the magnetic field strength and gradient decrease, thus a better temperature uniformity can be achieved (Figure 9c–e). Based on the simulation results and preliminary experimental trials, the induction heating process development was conducted within 2–3 kW generator power range, for 20–45 mm standoff distances, aiming to identify the optimum trade-off between process parameters that promotes a temperature increase above the PEKK melting region and facilitates debonding, without specimen overheating. Stand-off distances below 20 mm, corresponding to magnetic field intensities in the range of 8–10 kA m^−1^, resulted in a steep temperature increase and specimen overheating; thus, they were not included in the analysis.

#### 3.4.2. Induction Heating Assessment

##### Reference Laminate Testing

**Figure 10:**
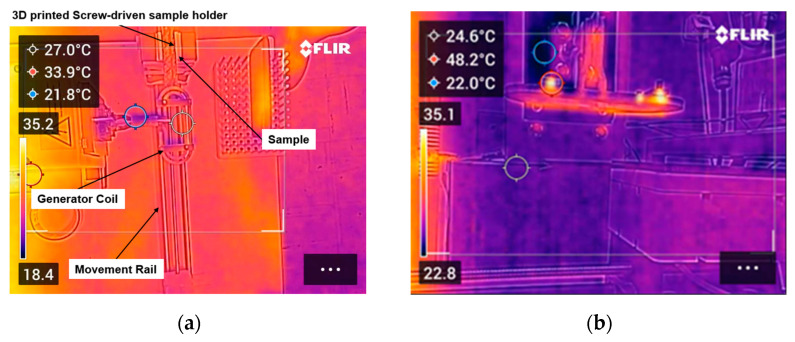
(**a**) Top-view image of the automated movement setup captured with the IR camera; (**b**) measurement of reference PAEK sample without nanocomposite FFF interlayer—no increase in specimen temperature recorded.

As a preparatory step prior to testing, reference PAEK laminates were investigated to confirm that they are electromagnetically inert within the range of experimental conditions tested and that no heating is induced by the application of the RF field. As shown in Figure 10b, no increase in specimen temperature was recorded, thus ensuring that induction heating only derives from the nanocomposite FFF interlayer.

##### Type I Specimens

Induction heating experiments of Type I specimens were performed at varying standoff distances (distance between the debonding zone and the coil) and power values to define the optimum process parameters that can facilitate debonding. In particular, standoff distance values ranged from 20 mm to 45 mm, while applied power values ranged from 2 kW to 3 kW. Initially, experiments were performed with regard to varying standoff distance values, while the applied power was maintained stable at 2 kW and 3 kW, respectively.

**Figure 11:**
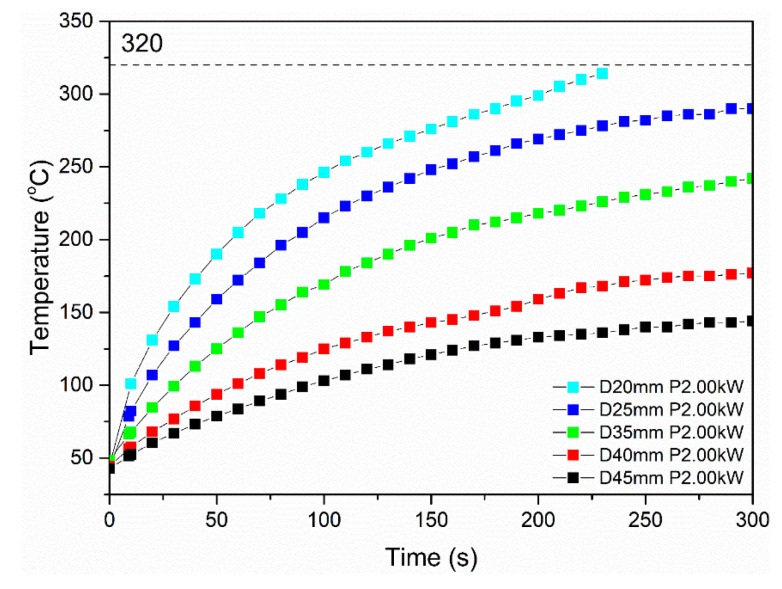
Representative induction heating curves of Type I specimens at 2 kW power value and varying standoff distance values (20–45 mm).

The induction heating results for the Type I specimens tested at 2 kW power and varying standoff distance values are presented in Figure 11. Therein, the impact of the standoff distance can be identified. In detail, the increase in the standoff distance limits the induction heating performance of specimens. Also, specimens tested at standoff distance values from 25 mm to 45 mm were heated at a steady rate, followed by various temperature plateaus at values lower than 300 °C. Considering that the specimen debonding is facilitated by the melting state of PEKK (T_m_ = 320 °C), the respective set of parameters cannot be applied for the debonding process. However, specimen testing at a standoff distance of 20 mm exhibited temperature increase at 320 °C after 220 s of measurement time, demonstrating enhanced induction heating capacity. This finding indicates that a standoff distance value of 20 mm, while applying 2 kW of power, can induce sufficient temperature increase in the debonding zone, so that the specimen can be debonded, using the continuous unzipping mechanism.

**Figure 12:**
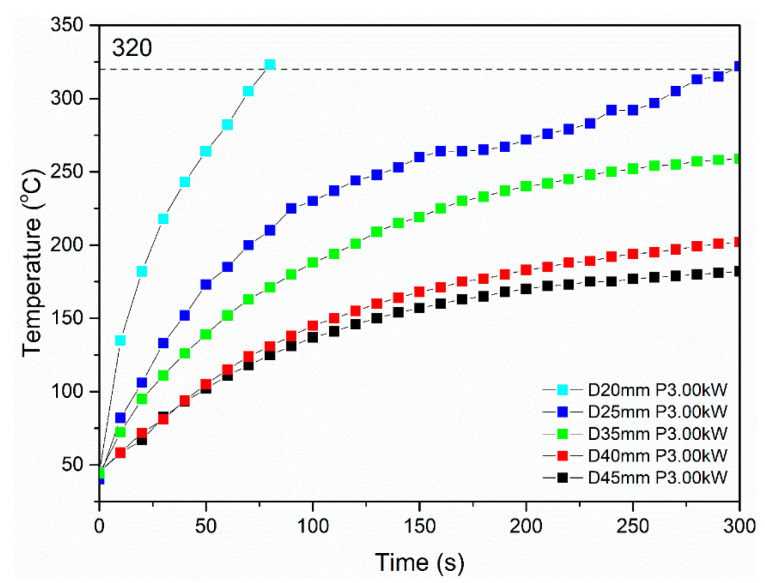
Representative induction heating curves of Type I specimens at 3 kW power value and varying standoff distance values (20–45 mm).

Experiments were repeated, with the same standoff distance values, while employing 3 kW of power. The respective induction heating results (Figure 12) indicate the same trend regarding the impact of the standoff distance in the heating performance of specimens. Despite the fact that the increase in power resulted in enhanced heating performance for all specimens, temperature plateaus were identified at values lower than 250 °C for specimens tested at standoff distance values ranging from 35 mm to 45 mm. Specimens tested at a standoff distance of 20 mm and 25 mm were heated above the melting temperature of pure PEKK (T_m_ = 320 °C), indicating that these sets of parameters can be applied in order to study the specimen debonding.

**Figure 13:**
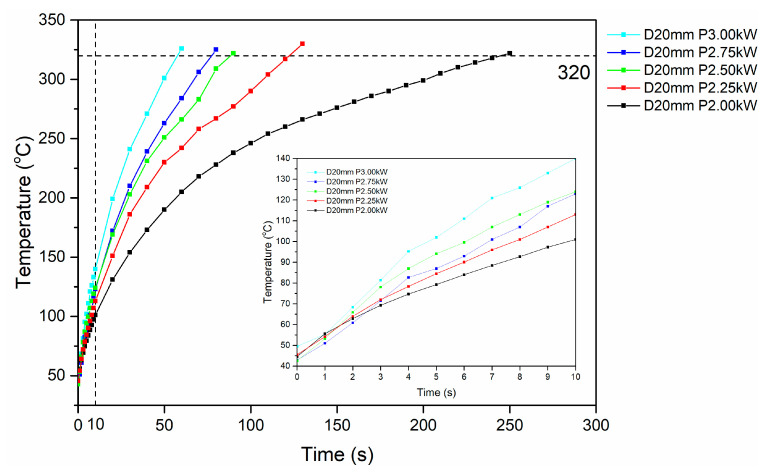
Representative induction heating curves of Type I specimens at 20 mm standoff distance and varying power values (2 kW–3 kW).

Collectively, results obtained from the induction heating experiments at power values of 2 kW and 3 kW, indicated that the specimens were effectively heated above the melting temperature of pure PEKK when the standoff distance was either 20 mm (for both 2 kW and 3 kW) or 25 mm (for 3 kW). For standoff distances over 35 mm, a temperature plateau is reached due to thermal losses by conduction into the specimen or by convection into the surrounding ambient air. As a standoff distance of 20 mm was critical in facilitating debonding in both cases of applied power, the process optimization was finalized with induction heating experiments conducted at a 20 mm standoff distance and varying power values ranging from 2 kW to 3 kW, with an increment of 0.25 kW per trial (Figure 13).

Based on the results of the induction heating experiments conducted at constant standoff distance and varying power values (Figure 13), it can be observed that all specimens were heated at temperatures higher than the melting temperature of pure PEKK, indicating that all sets of parameters can be applied for the investigation of specimen debonding. By adjusting generator power, initial heating rates within the range of 5.3–9.4 °C/s were achieved, with all specimens tested reaching the targeted temperature for debonding, i.e., above 320 °C. Specimens tested with power values from 2.5 kW to 3 kW were heated above 320 °C, within less than 90 s of RF field exposure, while specimens tested at 2 kW and 2.25 kW exceeded the specific temperature after 130 s and 250 s of measurement time, respectively (Table 5). Overall power values of 2.5, 2.75 and 3 kW presented similar performance for fast sample heating, while 2 and 2.25 kW values provide a slower heating rate that can facilitate heat dissipation and promote temperature uniformity. The high heating rates observed indicate that the degree of dispersion and agglomeration observed in Figure 5 induces a hysteretic heating behavior for the applied field intensity of c.a. 5 kAm^−1^ (Figure 9c).

##### Type II Specimens

Based on results obtained from induction heating assessment of Type I specimens, the process window of 2–3 kW power at 20 mm standoff distance presented a promising heating performance, with all sets of process parameters (standoff distance, power) being able to induce a sufficient temperature increase above the PEKK melting temperature range in the specimen debonding zone. Subsequently, the defined process window was further tested with Type II (gyroid) specimens to assess the effect of the FFF interlayer integration in a more complex geometry.

**Figure 14:**
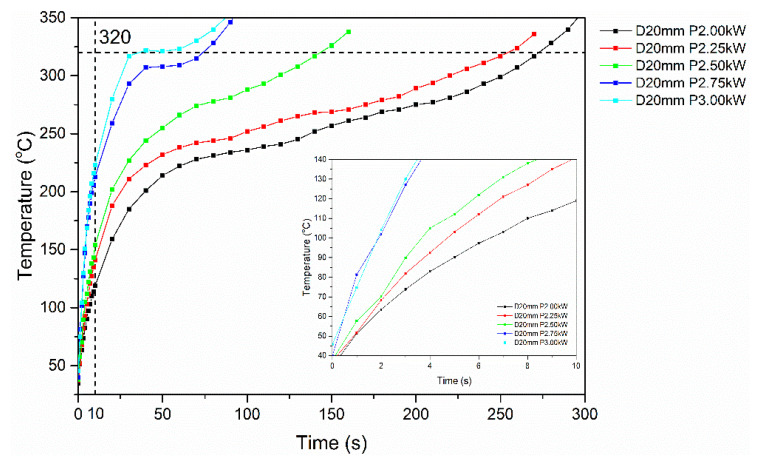
Representative induction heating curves of Type II (gyroid) specimens at 20 mm standoff distance and varying power values (2 kW–3 kW).

Based on the trial results presented in Figure 14, all specimens were heated above the melting temperature of PEKK (320 °C), with initial heating rates within the range of 8.0–17.5 °C/s; thus, all experimental conditions tested were within the debonding window definition for Type II specimens. The results for Type II specimens follow the same trend as in Type I, with 2 and 2.25 kW power values providing a slower heating rate and reaching debonding temperature after 250 s (Table 6). Further, specimens tested at 2.75 kW and 3 kW of power exhibited higher heating rates, reaching debonding temperature under 70 s of RF field exposure, indicating a larger influence of the power applied to the testing. For the equivalent FFF interlayer active surface area, higher initial heating rates were demonstrated in Type II specimens, possibly associated with the differences in magnetic flux density due to specimen geometry and the higher degree of compaction and lack of porosity observed in Type II specimens via mCT (Figure 8c,d). In addition, it should be noted that Type II specimens demonstrated temperature plateaus which, in contrast to the plateaus observed in Type I specimens as result of thermal losses, were followed by a steady temperature increase, which eventually exceeded the targeted debonding temperature. The presence of these plateaus can be attributed to the geometry and material characteristics of Type II gyroid specimens, as the gyroid structure consisting of pure PEKK is adjacent to the nanocomposite debonding area, which facilitates heat dissipation and possibly induces local endothermic phase changes in its proximity [45]. This effect becomes more pronounced in 3.00 and 2.75 kW power values, in temperatures near PEKK matrix melting region, where temperature plateaus appear at approximately 25 s and 40 s of measurement time, respectively. The results indicate that a high heating rate can be combined with a steady temperature time interval in which debonding can be conducted without compromising laminate quality. Although this effect could not be fully assessed due to lack of thermal insulation in the sample, it will further be studied and exploited in future experiments as a promising time frame for debonding.

##### Debonding of Type I and II Specimens and Inspection

Following the static induction heating trials in Type I and Type II specimens, the process window of 2–3 kW power at 20 mm standoff distance was further employed for debonding trials.

**Figure 15:**
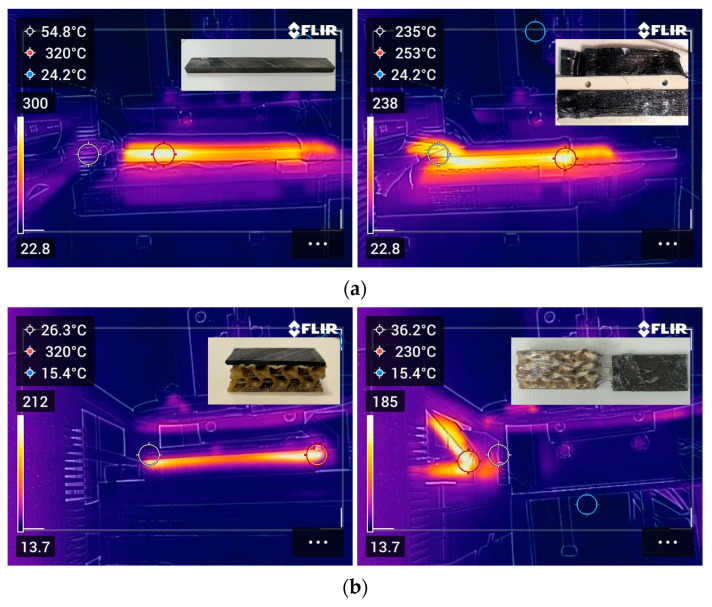
Indicative thermal camera images of debonding of Type I (**a**) and Type II (**b**) specimens, coupled with specimen structure prior and after debonding.

Specifically, each specimen was placed in the mounting setup as described in Section 2.4.1, and the debonding zone was carefully aligned with the ceramic blade position by adjusting the height of the latter. The mounting setup was set in linear motion when the recorded temperature reached 320 °C, and specimens were moved towards the ceramic blade at a constant linear speed of 2.0 mm/s (Figure 15). In all specimens tested (Type I and II), the full unzipping of the FFF debonding zone was achieved. Subsequently, CFRP laminate samples retrieved from debonded specimens were further analyzed to assess their quality and morphology of the nanocomposite layer.

**Figure 16:**
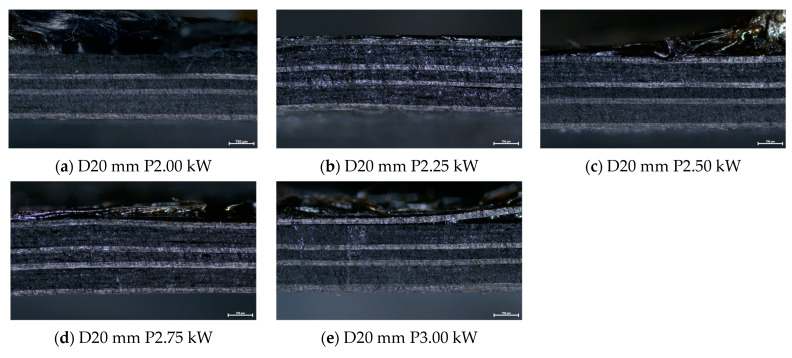
(**a**–**e**) Cross section images of debonded CFRP laminates extracted from Type I specimens processed under 2–3 kW generator power range at 20 mm standoff distance (scale bar: 750 μm).

**Figure 17:**
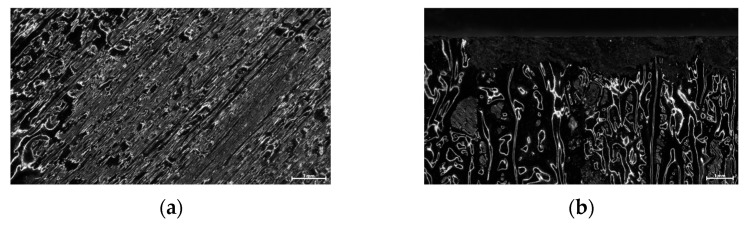
Top-view images of debonded surface morphology for 2 kW (**a**) and 3 kW (**b**) generator power at 20 mm standoff distance (scale bar: 1 mm).

**Figure 18:**
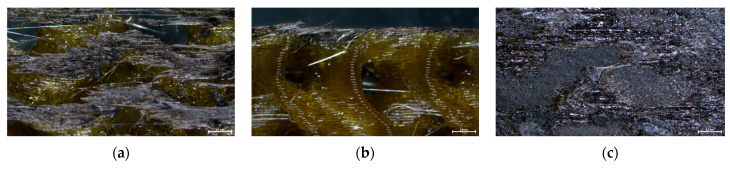
Top (**a**) and side (**b**) view of debonded gyroid structure and CFRP laminate (**c**) of Type II specimen processed under 2 kW generator power at 20 mm standoff distance (scale bar: 2.5 mm).

The cross sections of debonded CFRP laminates were inspected via optical microscopy to assess any visible indications of overheating and delamination. As observed in Figure 16, there is no evident impact on laminate quality when the applied power ranges from 2 to 2.5 kW, while delamination starts to occur for 2.75 kW and overheating becomes more pronounced for the 3 kW generator power. Additionally, the debonded surface morphology for the two marginal power values (2 kW and 3 kW) is depicted in Figure 17, where the effect of induction heating process conditions is observed in the resulting FFF debonding zone residues, and the surface texture transitions from rough to smooth and a fully melted state. In Figure 18, the respective debonded constituents of Type II gyroid specimen processed under a 2 kW generator power at a 20 mm standoff distance can be observed, where the residues of the FFF debonding zone can be seen around the previous contact points among the gyroid structure and CFRP laminate (Figure 18c). 

**Figure 19:**
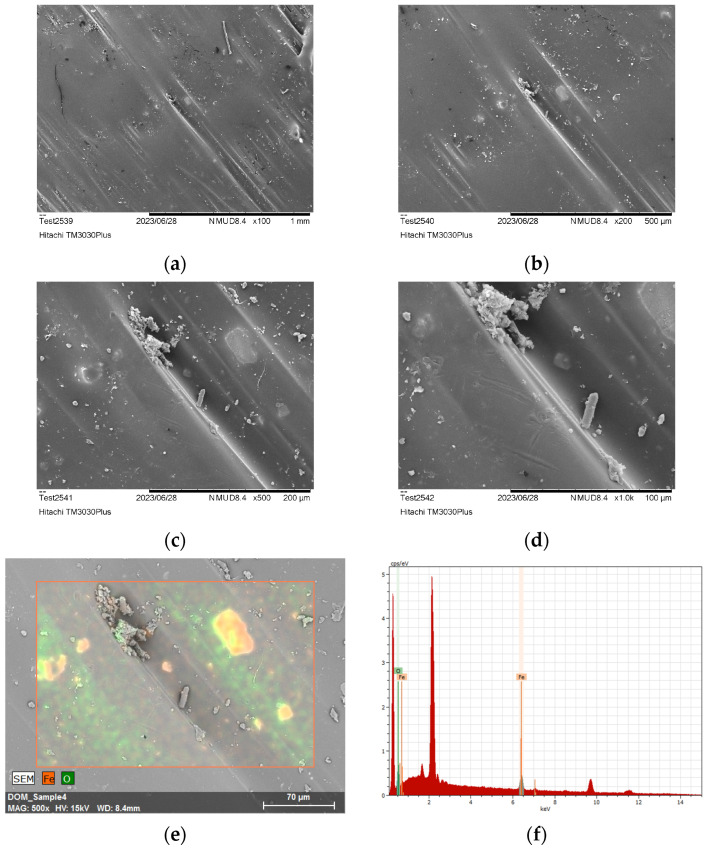
SEM morphology images (**a**–**d**) and EDS elemental analysis (**e**,**f**) of the intermediate layer (debonding zone) of debonded specimen.

SEM coupled with EDS analysis was conducted to further study the morphology of the nanocomposite layer and perform elemental analysis. EDS was employed to identify possible traces of the Fe_3_O_4_ agglomerates in the PEKK matrix of the debonding zone; Fe_3_O_4_ agglomerates were observed in all debonded surfaces with sizes ranging from 10 μm to 30 μm. This finding is also depicted in the EDS analysis (Figure 19f) through the distinct O and Fe peaks. It should be noted that peaks corresponding to approximately 2 keV represent the presence of Au traces, which is attributed to the sputter-coating of all specimens prior to SEM characterization. All debonded specimens exhibited surface transformations in the intermediate layer, which correspond to the melting of the nanocomposite layer. During the disassembly process, the nanocomposite layer may be completely or partially detached from the adjacent LM-PAEK CFRP layers due to the separation mechanism employed. A moderate contribution of the tape layup direction employed in AFP manufacturing can be observed in the surface morphology, with the directionality of the tape-laying morphology propagating from the CFRP laminate to the subsequent FFF interlayer (Figure 19a–d).

**Figure 20:**
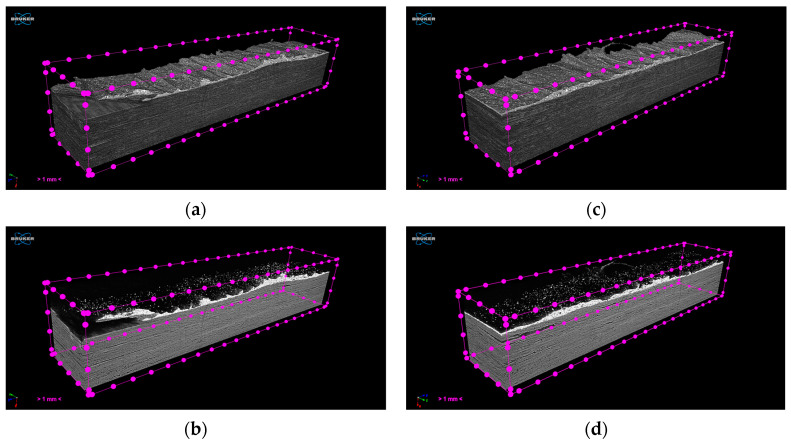
Three-dimensional visualization of CFRP laminates (**a** and **b**, **c** and **d**) and residual FFF nanocomposite interlayer after induction heating and debonding (white regions: NP agglomerates, gray region: CFRP/polymer matrix, black: background/air). Top (**a**,**c**)/bottom (**b**,**d**) images depict the same samples, with adjustment of the attenuation coefficient range to isolate features with different absorptivity (scale bar: 1 mm).

Finally, the assessment of the internal structure of debonded CFRP laminates was conducted via mCT scanning (Figure 20). Extracted CFRP laminates were scanned at 5.5 μm voxel size after induction heating treatment and debonding. The debonded surface morphology presents a wavy texture due to remelting and detachment, while a clean detachment without residue is observed at the corner of the specimen, indicating that the complete removal of the FFF interlayer is feasible upon further optimization of the ceramic blade positioning. In accordance with the results obtained through optical inspection (Figure 16), a small degree of delamination in PAEK CFRP is observed due to the heat treatment; however, the overall internal structural integrity is maintained.

## 4. Conclusions

In this study, localized induction heating and the debonding of sandwich-structured composite laminate panels were investigated in testing coupons prepared by AFP and extrusion-based AM technologies. By exploiting advanced composite manufacturing technologies, the integration of discrete debonding zones in two types of sandwich-structured laminate composites has been demonstrated. Induction heating was primarily enabled by the incorporation of nanocomposite interlayers (debonding zones) consisting of PEKK and Fe_3_O_4_ nanoparticles acting as electromagnetic susceptors. To investigate induction heating performance and debonding feasibility, a bespoke experimental setup was developed, allowing the optimization of the induction heating process parameters and effective separation of the different layers of laminate composites.

Based on the magnetic field simulation results and preliminary experimental trials, the induction heating process development was conducted within a 2–3 kW generator power range, for 20–45 mm standoff distances from the coil, corresponding to low magnetic field intensities in the range of 1–5 kA m^−1^. Simultaneous thermal imaging enabled the identification of the critical process parameters, aiming to achieve an optimum trade-off between a temperature increase above the melting point of pure PEKK (T_m_ = 320 °C), which would enable the effective melting of the debonding zone without specimen overheating. The results obtained indicated that the specimens were effectively heated above the melting temperature of pure PEKK when the standoff distance was 20 mm (for both 2 kW and 3 kW) or 25 mm (for 3 kW). By adjusting the generator power for a standoff distance of 20 mm, the initial heating rates within the range of 5.3–9.4 °C/s were achieved for Type I and 8.0–17.5 °C/s for Type II specimens, respectively. In both specimen types, a 2 kW power value provided a slower heating rate, reaching the debonding temperature after 250 s. All specimens were heated at temperatures higher than the melting temperature of pure PEKK, thus allowing the definition of the debonding process window that was further tested after static heating experiments.

For debonding trials, each specimen was placed in the mounting setup and the debonding zone was carefully aligned with a ceramic blade. The mounting setup was set in linear motion when the recorded temperature reached 320 °C, and specimens were moved at a constant linear speed of 2.0 mm/s. In all specimens tested (Type I and II), the full unzipping of the FFF debonding zone was achieved. Subsequently, CFRP laminate samples retrieved from debonded specimens were further analyzed to assess their quality and the morphology of the nanocomposite layer. The outcomes of this study provide an initial baseline for the development of rapid, on-demand joining, repair and disassembly technologies for thermoplastic composites towards new DfD strategies and more efficient maintenance, repair and overhaul operations. Future research directions should aim at the optimization of nanoparticle dispersion, further analysis of the dynamic rheological and thermal properties of the nanocomposite and the assessment of joint mechanical performance to establish reliable process–structure–property–performance relationships for the design and integration of debonding zones in more complex joint geometries. Additionally, the investigation of repair, remanufacturing and repurposing strategies of laminate composites to improve their circularity and performance should be a future research direction as well.

## Figures and Tables

**Table 1:** Extrusion conditions for filament production of PEKK nanocomposite with 7.5 wt% content of Fe_3_O_4_ nanoparticles.

Zone	2	3	4	5	6	7	8	Die	Melt Pump
Temp. (°C)	290	320	325	330	335	335	340	340	335
Screw Speed (rpm)		340	Melt Pump Speed (rpm)		15	Gravimetric Feeder Speed (rpm)			10

**Table 2:** Type I and II sandwich-structured composite laminate specimen manufacturing.

	Type I Specimens	Type II Specimens
Process sequence	AFP → FFF → AFP	AFP → FFF → FFF
Cross section schematic	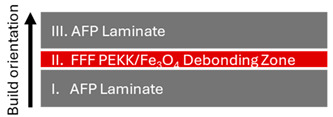	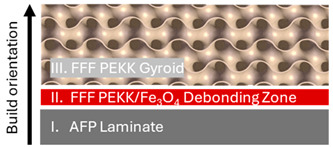
Cross section image (scale bar: 2.5 mm)	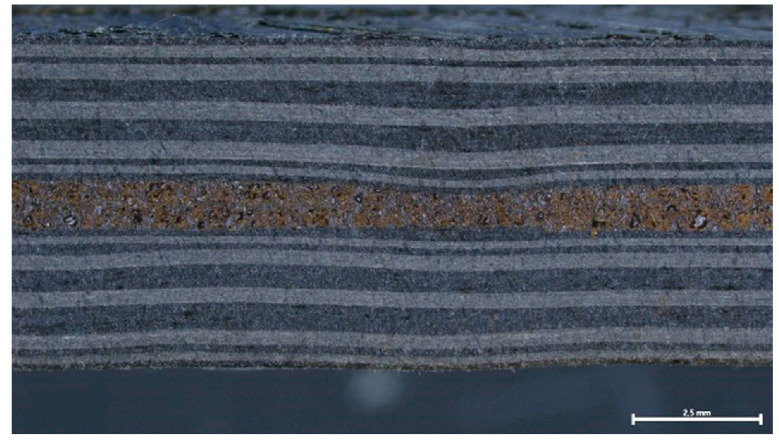	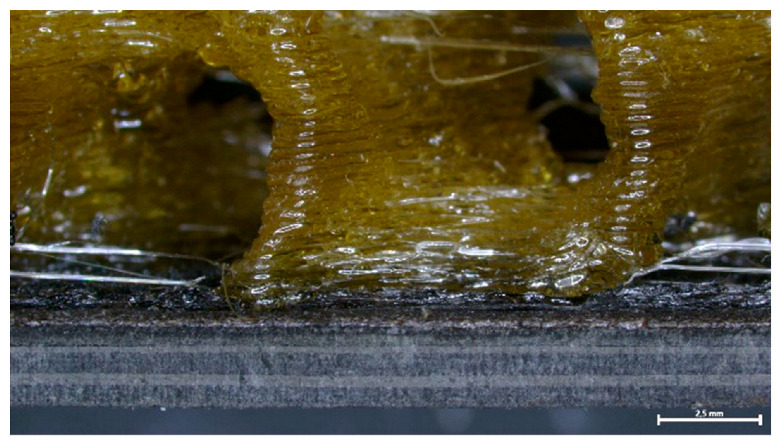
Design	AFP laminate stacking sequence: [45/0/135/0/0/135/90/45/0]s Consolidated AFP laminate thickness: 2.7 mm FFF debonding zone thickness: 0.6 mm	AFP laminate stacking sequence: [−45/45/0/0/90/90/45/−45]s Consolidated AFP laminate thickness: 2.45 mm FFF debonding zone thickness: 0.6 mm FFF gyroid lattice unit size: 15 mm
W × D × H	100 mm × 20 mm × 6 mm	80 mm × 25 mm × 18 mm

**Table 3:** Induction heating process parameters.

Control Factor	Units	Levels					Response Variables
Generator power (P)	kW	2	2.25	2.5	2.75	3	▪ Heating rate ▪ Time to reach debonding temperature > 320 °C
Frequency	kHz	415	414	414	413	412	
Current (rms)	A	302	322	341	350	376	
Voltage	V	354	377	400	413	442	
Standoff distance (D)	mm	20	25	35	40	45	

**Table 4:** DSC estimated values of thermodynamic quantities and TGA thermal degradation onset temperature for PEKK and 7.5 wt% samples at different processing stages. Crystallization temperature (T_c_) and enthalpy (ΔH_c_), glass transition temperature (T_g_), cold crystallization temperature (T_cc_) and enthalpy (ΔH_cc_), melting peak temperature (T_m_) and enthalpy (ΔH_m_), onset decomposition temperature (T_o/95%_) corresponding to 5% weight loss of the initial mass.

Sample	DSC Scan 2							TGA
	Cooling		Heating					
	T_c_ (°C)	ΔH_c_ (J/g)	*T*_g_ (°C)	*T*_cc_ (°C)	Δ*H*_cc_ (J/g)	*T*_m_ (°C)	Δ*H*_m_ (J/g)	T_o/95%_ (°C)
S1	-	0	159	262	5	307	6	520.5
S2	221	1	159	261	4	306	7	529.6
S3	-	0	159	263	3	307	4	523.2

**Table 5:** Initial heating rate over 0–10 s and time to reach debonding temperature for 20 mm standoff distance within 2–3 kW power range tested with Type I specimens.

Type I/D = 20 mm	P = 2.00 kW	P = 2.25 kW	P = 2.50 kW	P = 2.75 kW	P = 3.00 kW
Initial heating rate [t = 0–10 s] (°C/s)	5.3 ± 0.3	6.5 ± 0.2	8.0 ± 0.4	7.9 ± 0.3	9.4 ± 0.4
Time to reach debonding T > 320 °C (s)	250	130	90	80	60

**Table 6:** Initial heating rate over 0–10 s and time to reach debonding temperature for 20 mm standoff distance within 2–3 kW power range tested with Type II specimens.

Type II/D = 20 mm	P = 2.00 kW	P = 2.25 kW	P = 2.50 kW	P = 2.75 kW	P = 3.00 kW
Initial heating rate [t = 0–10 s] (°C/s)	8.0 ± 0.5	10.2 ± 0.5	11.1 ± 0.7	16.5 ± 1.5	17.5 ± 1.3
Time to reach debonding T > 320 °C (s)	270	250	140	70	40

## Data Availability

The original contributions presented in the study are included in the article; further inquiries can be directed to the corresponding author/s.

## Appendix A

For the development of the computational model, the following governing equations were used:

(A1) ∇×H=J

(A2) B=∇×A

(A3) E=jωA

(A4) J=σΕ+jωD

where ***H*** is the magnetic field intensity, ***E*** is the electric field strength, ***B*** is the magnetic flux density, ***J*** is the current density vector, σ is the electrical conductivity and ***A*** is the magnetic vector potential. The set of equations is completed by the following relations:

(A5) $$B=\mu_{0}\mu_{r}H$$

(A6) $$D=\varepsilon_{0}\varepsilon_{r}E$$

where *μ_r_* is the relative permeability and *ε_r_* is the relative permittivity. A bounding box around the experimental setup is selected to model the surrounding air around the geometry, and a magnetic insulation boundary condition is imposed on the bounding box boundaries:

(A7) n×A=0

The size of the bounding box size was determined by increasing the box size until the model results are unaffected by further size increase (Figure A1a). The coil was simulated by imposing the respective experimental conditions of Table 3. The conductivity and permittivity of the different materials was taken from the experimental measurements and the equipment manufacturer data sheet. A computational mesh of 197,247 elements was used to discretize the computational domain, and a quadratic basis function was used for the dependent variables (Figure A1b). The set of equations was solved using COMSOL Multiphysics.
